# Optimization of time and energy in straight one-sided robotic assembly lines

**DOI:** 10.1038/s41598-025-94202-y

**Published:** 2025-04-17

**Authors:** Rehab Seif ElMolouk, Amin M. K. El-Kharbotly, Raghda B. Taha

**Affiliations:** 1 Department of Design and Production Engineering, Faculty of Engineering, Ain-Shams University, Cairo, Egypt; 2 College of International Transport and Logistics, Arab Academy for Science, Technology and Maritime Transport, Cairo, Egypt

**Keywords:** Robotic assembly line balancing, Energy efficient assembly line, Multi-objective optimization, Mechanical engineering, Electrical and electronic engineering

## Abstract

Robotic assembly lines serve as a foundational element of modern manufacturing, facilitating the efficient production of high-quality goods. Reducing the energy consumption of robots in these assembly lines is essential to promoting greener manufacturing practices, lowering costs, and achieving global energy efficiency goals. This study seeks to create a model that optimizes robotic assembly line systems by minimizing cycle time and energy consumption, either independently or simultaneously. The research assumes an unlimited supply of various robot types, each with distinct variants, processing times, and energy demands for specific tasks. The problem is modeled using Integer Linear Programming (ILP) in the LINGO (21) solver. For multi-objective scenarios involving both cycle time and energy consumption, a weighted sum approach is applied to convert the problem into a single-objective format. To tackle large-scale problems more effectively, several concepts and rules are proposed to accelerate data processing. The results demonstrated improved performance compared to benchmark problems. The analysis indicated that reducing cycle time contributes to lower energy consumption, driven by an increase in the number of stations and robots. Additionally, the Pareto front analysis of cycle time and energy consumption revealed that energy usage remains nearly constant across a wide range of cycle times.

## Introduction

Manufacturing industries focus on reducing energy consumption due to rising costs and environmental concerns. The use of robots in manufacturing enhance speed and accuracy while minimizing failures, cycle times, and energy usage, as noted by Sadek et al.^1^, Abdulkader et al.^2^, and Shakeri et al.^3^.

Robotic assembly lines have revolutionized industrial manufacturing by introducing a range of advantages that significantly enhanced productivity, quality, and overall operational efficiency. Proper robot-task allocation is essential for optimal performance.

Lahrichi et al.^4^, Li et al.^5^, Gao et al.^6^, and Mukund Nilakantan et al.^7,8^ investigated straight robotic assembly line balancing, primarily minimizing cycle time. Lahrichi et al.^4^ examined robot assignment constraints, while Li et al.^5^ used CPLEX for small problems and simulated annealing (RSA) for larger ones. Gao et al.^6^ employed a hybrid GA-local search method. Mukund Nilakantan et al.^7^ added production rate maximization as a secondary objective, solving it with PSO, while Mukund Nilakantan et al.^8^ combined PSO and CS-PSO.

Çil et al.^9^ addressed mixed-model robotic assembly balancing using a beam search heuristic. Lopes et al.^10^ optimized cycle time in multi-manned robotic lines using mixed-integer linear programming. Belkharroubi et al.^11^ minimized energy consumption in multi-model lines with a Memory-Based Cuckoo Search Algorithm (MBCSA).

Li et al.^12^ minimized makespan in mixed-model assembly lines (RMALB/S) using CPLEX for small cases and metaheuristics for larger ones. Li et al.^13^ studied two-sided robotic lines, optimizing energy and cycle time with restarted simulated annealing.

Mukund Nilakantan et al.^14^, Sun et al.^15^ focused on energy and cycle time minimization in straight assembly lines. Mukund Nilakantan et al.^14^ optimized objectives separately using PSO, while Sun et al.^15^ used a multi-objective BHEDA algorithm.

Li et al.^16^ studied straight robotic assembly lines with NSGA-II and IMABC, optimizing cycle time alongside purchasing or production costs. Mukund Nilakantan et al.^17^ addressed straight and U-shaped lines, minimizing assemble line cost and cycle time with Differential Evolution. Zhang et al.^18^ reformulated a nonlinear model into a linear one, solving it with a Pareto artificial bee colony algorithm (PABC). The model optimized cycle time and energy consumption in U-shaped lines. Rabbani et al.^19^ solved mixed-model U-shaped balancing with NSGA-II and MOPSO, considering robot costs and cycle time.

Levitin et al.^20^ used Genetic Algorithms to reduce cycle time. Renna^21^ introduced fractional tasks and parallel workstations to mitigate failures, solved via metaheuristics. Zacharia and Nearchou^22,23^ optimized fuzzy cycle time and smoothness in type-2 assembly lines and ALWABP using Multi-Objective Genetic Algorithms. Zhou and Wu^24^ minimized workstations and energy in sustainable robotic assembly lines using MOEA/D.

For two-sided robotic assembly lines: Huang et al.^25^ optimized energy and cycle time using Simulated Annealing. Wu et al.^26^ minimized cycle time and fuel consumption in JRALB-FP using KEDA. Yadav and Agrawal^27^ maximized workstation workload via Branch and Bound. Li et al.^28^ used CPLEX and Co-Evolutionary Particle Swarm Optimization to minimize cycle time. Aghajani et al.^29^ minimized cycle time in mixed-model lines using SA.

Li et al.^30^ optimized cycle time and carbon footprint in cross-station RALBP via PSO. Chica et al.^31^ applied MOEAs to optimize station count, cycle time, and space in r-TSALBP. Calzavara et al.^32^ optimized makespan and human energy in collaborative assembly. Wang and Jiao^33^ incorporated human trust in task allocation. Jiao et al.^34^ optimized two-sided U-shaped collaborative lines using a heuristic algorithm.

A summary of the literature on robotic assembly line balancing is presented in Table 1.

**Table 1:** The robotic assembly line literature.

References	Type				Objective					No of robots		Solution method	
	Straight	Two sided	u shaped	Mixed	Cycle time	Energy consumption	Prod. rate	Smoothness index	Prod. cost	1	More than 1	Exact	Algorithm or Heuristic
Lahrichi et al. ^4^	√				√					√			√
Li et al. ^5^	√				√					√		√	√
Gao et al. ^6^	√				√					√			√
Mukund Nilakantan et al. ^7^	√				√		√			√			√
Mukund Nilakantan et al. ^8^	√				√					√			√
Çil et al. ^9^	√			√	√								√
Lopes et al. ^10^	√				√						√	√	
Belkharroubi et al. ^11^				√		√				√			√
Li et al. ^13^		√			√	√				√			√
Mukund Nilakantan et al. ^14^	√				√	√				√			√
Sun et al. ^15^	√				√	√				√			√
Li et al. ^16^	√				√				√	√			√
Mukund Nilakantan et al. ^17^	√		√		√				√	√			√
Zhang et al. ^18^			√		√	√				√			√
Rabbani et al. ^19^			√	√	√				√	√			√
Levitin et al. ^20^	√				√					√			√
Huang et al. ^25^		√			√	√				√			√
Li et al. ^28^		√			√					√		√	√
Aghajani et al. ^29^		√		√	√					√			√

The literature review identified two distinct variants of robotic assembly lines based on robot availability and allocation. In the first variant, robots from a given set can be assigned to multiple workstations without any restrictions. In the second variant, each robot is restricted to at most one workstation. Furthermore, most studies in this field focus on optimizing cycle time as a single objective. Research addressing other objectives—such as energy consumption, makespan cost, workload distribution, efficiency, and carbon footprint—either individually or simultaneously, remains relatively scarce. This is largely due to the complexity of handling multiple objectives in large-scale problems, which are classified as NP-hard. As a result, in multi-objective optimization problems, researchers typically either tackle each objective separately or employ meta-heuristic techniques to derive solutions.

This research aims to develop an Integer Linear Programming (ILP) model to optimize cycle time and energy consumption for a one-sided straight robotic assembly line, both individually and using a weighted sum approach. The study follows the second variant of the assembly line (rALB-II), where each robot type can be allocated to multiple workstations without constraints. Various problem sizes and workstation counts are considered. The model’s performance is assessed by comparing the results with published benchmark problems. Single-objective problems, focusing on either cycle time or energy consumption, are solved using linear programming, complemented by heuristics for large-scale problems. Multi-objective optimization is addressed through the weighted sum method. Additionally, a Pareto front is generated for cycle time and energy consumption to visualize trade-offs, facilitating an evaluation of resource allocation efficiency and overall outcomes.

## Mathematical model and solution method

### Nomenclature

Indices

- *i, j*: Index of assembly tasks, *i, j* = 1, 2, …, *Na*
- *r*: Index of robot types, *r* = 1, 2, …, *Nr*
- *s*: Index of workstation, *s* = 1, 2, …, *Nw*

Parameters

- $N_{a}$: total number of tasks
- $N_{r}$: total number of robots
- $N_{W}$: total number of workstations (robots) where $N_{r} = N_{W}$
- $T_{ir}$*:* processing time of task *i* by robot type *r*
- $T_{s}$: total execution time for workstation s
- pre(*i*): set of immediate predecessors of task *i*
- $EP_{r}$: the energy consumed by robot *r* per time unit on processing time
- *E*_*ir*_: the energy needed for processing task i by robot type *r*
- $E_{r}$: the total energy consumed.
- CT: Cycle time

Decision variables

- $$X_{is} = \left\{ {\begin{array}{*{20}l} 1 \hfill & {\quad {\text{if task i is assigned to workstation s}}} \hfill \\ 0 \hfill & {\quad {\text{otherwise}}} \hfill \\ \end{array} } \right.$$
- $$Y_{sr} = \left\{ {\begin{array}{*{20}l} 1 \hfill & {\quad {\text{if robot r is allocated to workstation s}}} \hfill \\ 0 \hfill & {\quad {\text{otherwise}}} \hfill \\ \end{array} } \right.$$

### The developed mathematical model

This study examines the robotic straight one-sided assembly line problem (rALB-II), which involves assigning $N_{a}$ tasks to $N_{W}$ workstations and allocating $N_{r}$ robots to these workstations. The primary goal is to minimize either cycle time or energy consumption, or both. It is assumed that $N_{a}$ tasks are distributed across $N_{W}$ workstations, with $N_{r}$ robots assigned accordingly. Tasks are performed sequentially, adhering to predefined precedence constraints. Each task ***i*** is completed within a specific time (*T*_*ir*_) using a designated type of robot. The time and energy consumption data are derived from studies conducted by Gao et al.^6^ and Mukund Nilakantan et al.^14^.

The model is based on the following assumptions, as stated in Levitin et al.^20^ and Gao et al.^6^:

- Tasks can be assigned to any workstation, provided precedence constraints are met.
- Each workstation must complete its assigned tasks within the given cycle time.
- There is no limitation on the total number of robots available for assignment.
- Each workstation is assigned exactly one robot, and each task is allocated to a single workstation $N_{r} = N_{W}$.
- The time and energy consumption for each task depend on the type of robot assigned.
- Precedence constraints must be maintained to ensure tasks are assigned and executed in the correct order.
- Task processing times are deterministic.
- Tasks cannot be further subdivided.
- Energy consumed during maintenance time is disregarded.
- The energy required for a task is determined by the type of robot used and the task’s time requirements, as represented in Eq. (1).
  1 $$E_{ir} = EP_{r} \cdot T_{ir}$$
  where $EP_{r}$ is the energy consumed by robot *r* per time unit on processing time.

#### Single objective model

The primary objective of this model is to minimize the cycle time while adhering to the precedence relationships and ensuring all model constraints are met as shown in Eqs. (2) and (3). The energy objective is shown in Eqs. (4) and (5).

##### Objective function: minimize the cycle time

2 $$Z_{1} = {\text{Minimize}}\;\;CT$$

where the cycle time is defined as the maximum total time required by any workstation. This equation is non-linear because it involves the maximum function and the product of decision variables ${\text{X}}_{{{\text{is}}}}$ and ${\text{Y}}_{{{\text{sr}}}}$

3 $$CT = \mathop {\max }\limits_{{1 < s < {\text{N}}_{{\text{w}}} }} \left\{ {\mathop \sum \limits_{{{\text{i}} = 1}}^{{{\text{N}}_{{\text{a}}} }} \mathop \sum \limits_{{{\text{r}} = 1}}^{{{\text{N}}_{{\text{w}}} }} {\text{X}}_{{{\text{is}}}} {\text{Y}}_{{{\text{sr}}}} {\text{T}}_{{{\text{ir}}}} } \right\}$$

##### Objective function: minimize the total energy consumed

4 $${\text{Z}}_{{2}} = {\text{Minimize}}\;\;E_{r}$$

where

5 $$E_{r} = \mathop \sum \limits_{i = 1}^{{N_{a} }} \mathop \sum \limits_{r = 1}^{{N_{r} }} \mathop \sum \limits_{s = 1}^{{N_{w} }} X_{is} Y_{sr} T_{ir} EP_{r}$$

where $T_{ir}$ is the processing time of task *i* performed by robot *r*, and $EP_{{\text{r}}}$ denotes energy consumed by robot *r* per time unit on processing time.

#### The multi-objective model

Both energy consumption and cycle time are optimized simultaneously using a weighted sum approach, as described by Zacharia and Nearchou^22^. The model accounts for precedence relationships while ensuring compliance with all constraints.

To enhance computational efficiency, particularly in the multi-objective optimization of large-scale problems, the following concepts and rules may be useful.

- The two objectives are combined into a single objective function using different weight ratios, as shown in Eq. (6). These ratios represent the relative importance of one objective over the other. Therefore, the problem is solved as a single-objective optimization at varying cycle time and energy consumption ratios. In this study, a 0.5:0.5 ratio is considered.
- Robots are assigned to stations by first treating only the robot assignment variables as integers. These variables are solved and fixed before setting the task assignment variables as integers. Once the robot allocation is determined, tasks are assigned to stations, following a standard assembly line balancing approach.
- To execute the model multiple times for different cycle time and energy ratios, the problem is initially solved with a small coefficient for energy in the objective function. The resulting cycle time and energy consumption values serve as the starting solution for cases where cycle time has a higher weight, as increasing the cycle time percentage typically increases the solving time.
- For larger problem instances, the relative optimality tolerance is set to 0.01, ensuring that the obtained solution remains within 1% of the true optimal value.
- In very large-scale problems, if solving time remains excessive after obtaining an initial solution for cycle time and energy consumption, the robot plantation concept (also known as robot fixation) is applied. This technique involves pre-assigning specific robots to workstations, restricting the placement of their predecessors and successors to streamline computation.
- Since robot fixation considers precedence constraints for both the earliest and latest workstations, it produces a near-optimal solution rather than an exact one.

**Objective Function**: Combined multi-objective function:

6 $${\text{Minimize}} \quad Z_{3} = w_{1} \cdot CT + w_{2} E_{r}$$

where *w*_*1*_ and *w*_*2*_ are weights for the objectives.

#### Model constraints

The model constraints are shown in Eqs. (7)–(9) while Eqs. (10)–(14) is added to linearize the cycle time objective present in Eq. (3)

- The precedence relationship between the tasks

7 $$\mathop \sum \limits_{s = 1}^{{N_{w} }} s X_{is} - \mathop \sum \limits_{s = 1}^{{N_{w} }} s X_{js} \le 0\quad \forall_{i} \in pre\left( j \right)$$

To ensure that for each pair of tasks with a precedence relationship, a successor task cannot be assigned until its preceding task has been assigned.

- Assignment each task to one workstation only

8 $$\mathop \sum \limits_{s = 1}^{{N_{w} }} X_{is} = 1\quad \forall_{i}$$

To ensure that each task is assigned to only one station.

- Each workstation is equipped with one robot.

9 $$\mathop \sum \limits_{r = 1}^{{N_{w} }} Y_{sr} { = 1}\quad \forall_{{\text{s}}}$$

To address the non-linearity in the assembly line balanng problem observed in Eq. (3), two auxiliary decision variables are added to the model. The first variable is *CT* to represent the maximum cycle time and second auxiliary variable is *Z*_*isr.*_ to represent the product of the two variables $X_{is} Y_{sr}$. This transformation involved incorporating an additional constraints shown in Eqs. (10)–(14) into the model to linearize the maximum operator and the quadratic term. This approach allows the problem to be solved as Integer Linear Programming and to handle the quadratic components and maximum operator effectively to achieve optimal solutions.

To ensure the linearization of objective function shown in Eq. (3).

10 $$Z_{isr} = X_{is} Y_{sr}$$

11 $$Z_{isr} \le X_{is} \qquad \forall_{{{\text{i}},{\text{s}},{\text{r}}}}$$

12 $$Z_{isr} \le Y_{sr} \qquad \forall_{{{\text{i}},{\text{s}},{\text{r}}}}$$

13 $$Z_{isr} \ge X_{is} + Y_{sr} - 1\qquad \forall_{{{\text{i}},{\text{s}},{\text{r}}}}$$

14 $$\mathop \sum \limits_{i = 1}^{{N_{a} }} \mathop \sum \limits_{r = 1}^{{N_{w} }} Z_{isr} T_{ir} \le CT\qquad \forall_{{\text{s}}}$$

## Results and discussion

The results obtained in the present work from minimizing cycle-time and/or energy consumption are compared with the best results reached by other researchers for certain benchmark problems found in literature^14,15,35^. The results of the proposed model for the two objectives are then discussed.

### Results of robotic straight assembly line balancing for optimal cycle time objective

The results obtained from applying the proposed model are compared to the benchmark problems available in literature. According to^4,5^, the robotic assemble line balancing problem may follow one of the two variants based on the concept of assigning robots to stations. The first variant forces each type of robot to be assigned to at most one workstation. While in the second variant, each type of robot can be assigned to multiple workstations without any limitation.

A sample benchmark problem (Problem 25-6) described by Zixiang Li^5^ is solved and analyzed. This problem’s precedence diagram comprises 34 direct precedence relations among 25 assembly tasks. Table 2 provides task assignments and robot allocations obtained using the proposed model with LINGO (21) solver.

**Table 2:** Solutions for the 25-6 problem.

Proposed model results						
	Workstation 1	Workstation 2	Workstation 3	Workstation 4	Workstation 5	Workstation 6
Task assignment	1,2,3	4,5,6,7	8,11,12,15	9,10,13,17	14,19,20,21,24	16,18,22,23,25
Total operation times	138	185	194	185	185	194
Robot allocation	R3	R3	R2	R5	R3	R4
Cycle time	194					

The results of 17 test problems are summarized in Table 3. Column I lists the problem numbers, while Column II shows the task sizes of the evaluated problems. Column III specifies the number of robots/workstations for each problem. Column IV presents the cycle time results for 10 problems solved using CPLEX under the first variant, as described by Gao et al.^6^. Column V provides the results obtained by Gao et al.^6^ using a hybrid GA with local search for the first variant, which assumes that only one robot is available for each robot type. Although their model differs in this respect, the hybrid GA results are used to set up the upper bound. Due to the nondeterministic nature of the algorithm and the problem, each test was run 10 times, and most runs converged to the same solution.

**Table 3:** Results of solving benchmark rALB problems.

Problem number	No. of tasks	No. of robots (stations)	1st variant (rALB-I)			2nd variant (rALB-II)				
			CPLEX ^6^	Hybrid GA ^6^	Split algorithm ^4^	Split algorithm ^4^	CS-PSO ^8^	PSO ^8^	BHEDA ^15^	Proposed model
(I)	(II)	(III)	(IV)	(V)	(VI)	(VII)	(VIII)	(IX)	(X)	(XI)
1	25	6	200	213	213	194	200	221	194.25	194
2	35	4	341	449	450	341	341	341	**–**	321
3		5	329	344	344	329	332	357	328.2	329
4		7	201	222	222	201	211	226	**–**	201
5		12	106	113	112	93	103	105	97.03	98
6	53	5	449	554	554	449	449	454	412	412
7		10	221	230	230	203	221	224	216.05	218
8		14	142	162	158	134	142	146	**–**	134
9	70	7	394	449	451	N/A	430	446	–	419
10		10	245	272	271	N/A	264	259	–	252
11		14	N/A	204	199	N/A	194	194	174	170
12		19	N/A	154	151	N/A	140	139	122.13	121
13	89	8	N/A	494	N/A	N/A	460	464	–	450
14		12	N/A	370	N/A	N/A	320	317	300.31	305
15		21	N/A	205	N/A	N/A	219	219	–	180
16	297	29	N/A	430	N/A	N/A	394	428	–	370
17		38	N/A	344	N/A	N/A	305	295	256.31	290

Column VI displays the cycle time results achieved by Youssef Lahrichi et al.^4^ whose model optimized cycle time using a “split” algorithm embedded within a metaheuristic framework to explore the space of giant sequences. They examined both robot assignment variants, with results for the second variant shown in Column VII. Mukund Nilakantan et al.^8^ addressed the second variant problem using a hybrid cuckoo search and particle swarm optimization (CS-PSO) approach, with results provided in Column VIII. Mukund Nilakantan et al.^8^ proposed a particle swarm optimization (PSO) algorithm for the second variant problem, developing both time-based and energy-based models, with the time-based results displayed in Column IX.

Sun et al.^15^ tackled the second variant problem using a multi-objective population-based evolutionary algorithm, BHEDA, with results presented in Column X. These results were extracted using WebPlotDigitizer, as the paper only provided a graphical representation.

The results achieved using the proposed model (Column XI) consistently outperform the PSO and CS-PSO solutions across all test instances. While CPLEX was able to find solutions for the first 10 problems, it failed to solve the remaining problems due to their NP nature. In contrast, the proposed model successfully provided solutions even for large-sized problems, aided by its innovative concepts and rules. The metaheuristic approach used by Lahrichi et al.^4^ was effective only for small-sized problems, requiring 30 runs to achieve satisfactory results.

### Results of robotic straight assembly line balancing for optimal energy objective

The proposed model has been applied to solve the benchmark problems presented by^15,35^ As illustrated in Table 4, the results demonstrate that the proposed model outperformed the benchmark solutions across all instances.

**Table 4:** Comparison of the results of balancing robotic assembly line for optimal energy with benchmark problems.

Problem	BHEDA energy ^15^	Energy proposed model
25-3	–	350
25-6	354	340
35-4	–	950
35-5	864	822
35-7	–	860.9
35-12	617	587
53-5	–	2550
53-10	2103.67	1876.1
53-14	–	1595.4

### Results of multi-objective balancing of robotic straight assembly line (*CT & Er*)

The proposed solution method addresses multi-objective robotic assembly line problems by optimizing both cycle time (*CT*) and energy consumption (*Er*). Problem sizes considered include P25, P35, P53, and P70, with varying numbers of workstations and robots analyzed using LINGO (21) solver. Results presented in Table 5 were derived by assigning equal weight values (0.5:0.5) to *CT* and *Er*. When the problem is solved as a multi-objective, the resulting cycle time is higher compared to solving it as a single objective focused solely on minimizing cycle time. This increase in cycle time is becoming more significant as the number of stations increases.

**Table 5:** Results of benchmark problems (weight ratio of CT:Er is 1:0, 0.5–0.5, and 0:1).

Problem number	No. of tasks	No. of stations/robots	WEST ratio	Results							
				CT (S.O.)	CT (M.O.) 0.5:0.5	∆ CT (VI-V)	∆ CT%	Er (S.O.)	Er (M.O.) 0.5:0.5	∆ Er (X-IX)	∆ Nr%
(I)	(II)	(III)	(IV)	(V)	(VI)	(VII)	(VIII)	(IX)	(X)	(XI)	(VIII)
1	25	6	4.16	194	205	11	5.67	394	350	44	12.57
2	35	4	8.75	321	337	16	4.98	1088	1021	67	6.56
3	35	5	7.00	329	331	2	3.29	995	874	121	13.84
4	35	7	5.00	201	252	51	25.37	1023	900	123	13.88
5	35	12	2.92	98	121	23	23.46	821	592	229	38.68
6	53	5	10.60	412	468	56	13.59	3249	2581.2	667.8	25.87
7	53	10	5.30	218	230	12	5.5	2355	1903	452	23.75
8	53	14	3.79	134	157	23	17.16	1745	1633	112	6.85
8	70	7	10.00	419	423	4	0.95	4031	3989.8	41.2	1.03
9	70	14	5.00	177	225	48	27.12	3371	2985	386	12.93

*S.O.* Single objective, *M.O.*  multi-objective, *∆CT *difference between cycle time, *∆Er* difference between energy.

In general, the WEST ratio significantly affects both cycle time and energy consumption. A lower WEST ratio corresponds to reduced cycle time and energy use. However, the extent of improvement in cycle time due to a lower WEST ratio depends on the problem size and the complexity of precedence relationships. This reduction in the WEST ratio is more impactful for larger problems. It was also observed that benchmark problems with the same number of tasks lack common task processing times, making direct comparisons challenging. When deciding to increase the number of stations to reduce the cycle time and energy, the associated investment and operational costs must be considered. Although robot idling energy is not accounted for, energy consumption decreases with an increased number of stations because tasks can be assigned to stations with robots that require less energy for specific tasks.

**Fig. 1:**
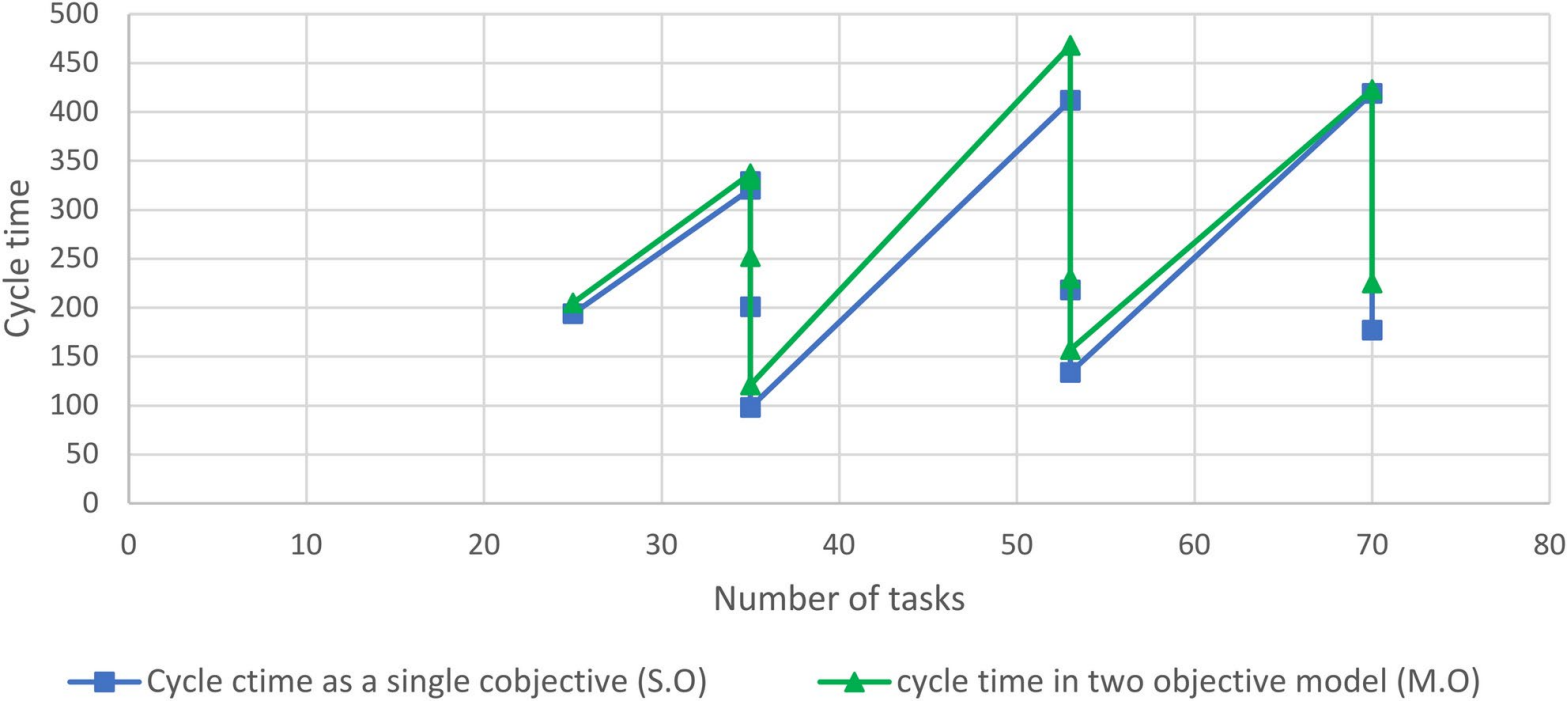
Comparing the results of single objective and multi-objective cycle-time.

Figure 1 illustrates that the increase in cycle time when applying the multi-objective model is influenced by the number of stations or the WEST ratio. A lower WEST ratio results in a higher percentage increase in cycle time, with the maximum increase observed for P70-14, reaching 27% at a WEST ratio of 5. This approach offers an attractive option for decision-makers, as it balances energy optimization with minimal impact on cycle time.

**Fig. 2:**
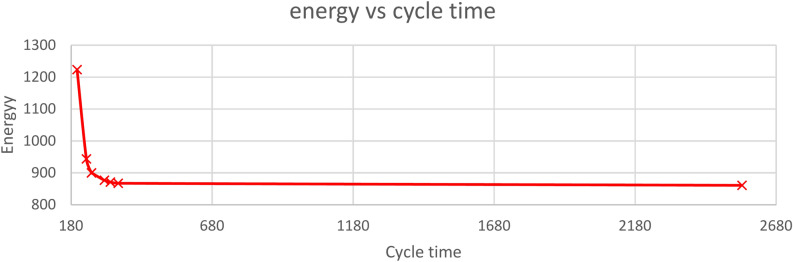
Pareto front for benchmark problem 35-7.

The proposed model was utilized to address the 35-7 problem by applying various weight combinations for cycle time (*CT*) and energy (*Er*), as detailed in Table 6. The resulting Pareto efficient frontier, depicted in Fig. 2, demonstrates that energy usage remains relatively stable across a wide range of cycle times. As shown in Table 7, both robot assignment and task allocation shift depending on the weights assigned to cycle time and energy. When optimizing solely for cycle time (ratio *Er:CT* = 0:1), tasks are assigned to robots without considering their energy consumption ratios. Specifically, 31 out of 35 tasks are allocated to robots with energy consumption ratios of 0.8 and 0.9, while only 4 tasks are assigned to robots with a 0.5 ratio. Furthermore, it is observed that these 31 tasks are distributed among robots 1, 2, and 6, with robot 2 handling 15 tasks and robot 6 managing 11 tasks, aiming to leverage the shorter processing times of these robots without breaching precedence constraints. Conversely, when optimizing solely energy (ratio *Er:CT* = 1:0), tasks are primarily assigned to robots with lower energy consumption ratios. In this scenario, 29 tasks are allocated to robots with a 0.5 energy consumption ratio, while 6 tasks are assigned to robots with ratios of 0.8 and 0.9. A similar task assignment pattern is observed, where the 29 tasks are distributed between robots 3 (17 tasks) and 7 (12 tasks). This behavior can be attributed to the same factors influencing task allocation during cycle time optimization. Increasing the number of stations could potentially reduce the number of tasks assigned to each station.

**Table 6:** The energy and cycle time for 35-7.

Cycle time weight	Energy weight	Cycle time	Energy
0	1	574	860.9
0.1	0.9	347	867.4
0.2	0.8	319	871.2
0.3	0.7	298	876.7
0.4	0.6	252	900
0.5	0.5	252	900
0.8	0.2	234	943.6
1	0	201	1023.8

**Table 7:** The task and robot assignment for 35-7.

	Station	Robot assignment	Tasks	Energy	Cycle time
Weighted sum ratio *(E*_*r*_*:CT) (0:1)*	Station 1	Robot 2 (0.8)	A,B,C,E,F,L	128.8	161
	Station 2	Robot 7 (0.5)	D,G,K,R	100.5	201
	Station 3	Robot 6 (0.8)	H,I,J,M,S	148	185
	Station 4	Robot 2 (0.8)	N,O,P,Q,T	157.6	197
	Station 5	Robot 2 (0.8)	U,V,BB,DD	154.4	193
	Station 6	Robot 6 (0.8)	W,X,Y,Z,AA,HH	153.6	192
	Station 7	Robot 1 (0.9)	CC,EE,FF,GG,II	180.9	201
				1023.8	201
Weighted sum ratio *(E*_*r*_*:CT) (0.5:0.5)*	Station 1	Robot 3 (0.5)	A,E,F,G,J	126	252
	Station 2	Robot 7 (0.5)	L,N,R,B,C	121	242
	Station 3	Robot 2 (0.8)	D,H,O,Q,S	144.8	181
	Station 4	Robot 3 (0.5)	I,P,T,U,V	114	228
	Station 5	Robot 7 (0.5)	K,W,X,Y,Z,AA	119.5	239
	Station 6	Robot 3 (0.5)	BB,DD,HH,M	117	234
	Station 7	Robot 2 (0.8)	CC,EE,FF,GG,II	158.4	198
				900.7	252
Weighted sum ratio *(E*_*r*_*:CT) (0.8:0.2)*	Station 1	Robot 3 (0.5)	A,B,E,F,G,J	153	306
	Station 2	Robot 7 (0.5)	L,N,H,C	172	86
	Station 3	Robot 2 (0.8)	Q,O,D	80.8	101
	Station 4	Robot 3 (0.5)	I,K,M,P,R,S	157	314
	Station 5	Robot 3 (0.5)	EE,DD,BB,V,U,T	159.5	319
	Station 6	Robot 7 (0.5)	FF,GG,Z,AA,W,X,Y	148.5	297
	Station 7	Robot 4 (0.9)	II,HH,CC	86.4	96
				871.2	319
Weighted sum ratio *(E*_*r*_*:CT) (1:0)*	Station 1	Robot 3 (0.5)	A,B,E,F,G,J	153	306
	Station 2	Robot 4 (0.9)	C,D	52.2	58
	Station 3	Robot 7 (0.5)	H,K,L,N	92	184
	Station 4	Robot 2 (0.8)	O,Q	45.6	57
	Station 5	Robot 3 (0.5)	I,M,P,R,S,T,U,V,BB,DD,EE	287	574
	Station 6	Robot 7 (0.5)	W,X,Y,Z,AA,FF,GG,HH	173.5	347
	Station 7	Robot 4 (0.9)	CC,II	57.6	64
				860.9	574

(0.x) = *P*_*r*_ which is ratio of power consumed by robot r relative to *CT.*

**Fig. 3:**
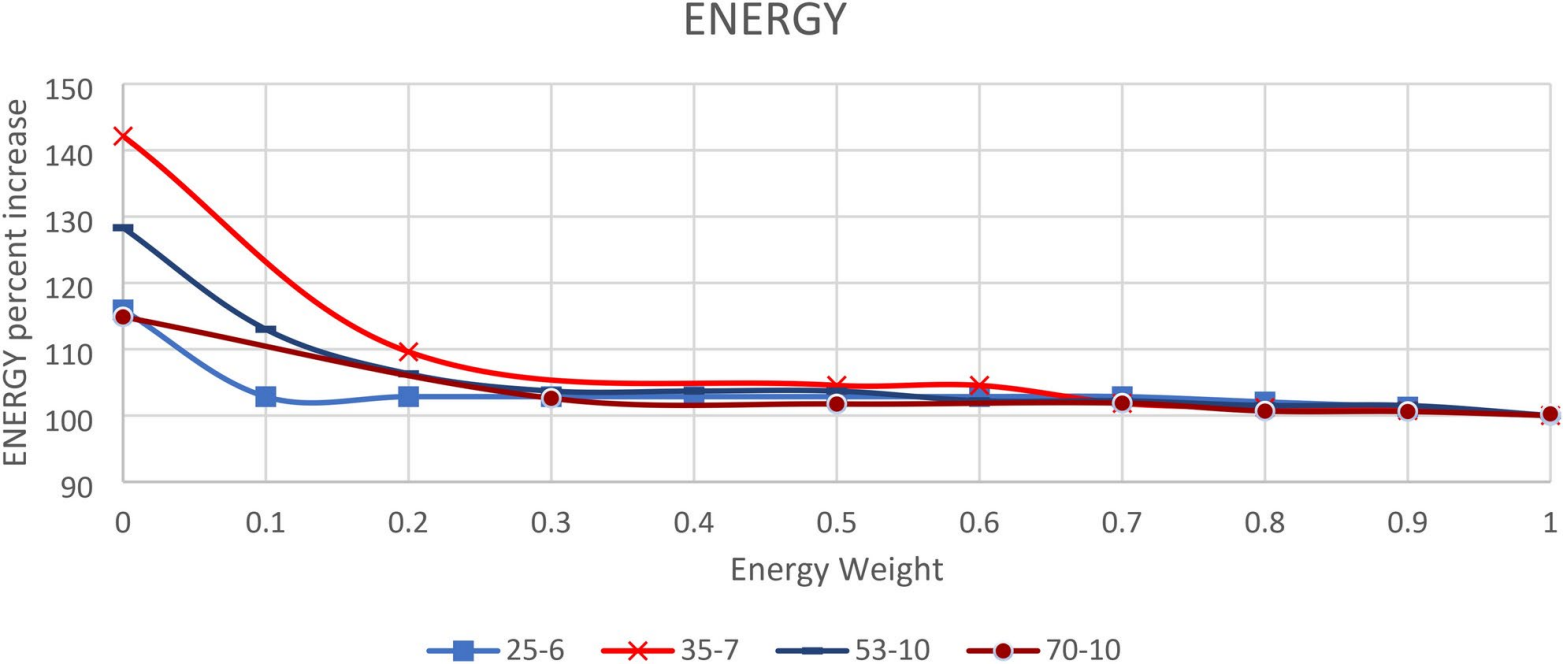
The percent decrease with Energy weight increase.

The benchmark problems under consideration vary in configuration, including the number of tasks, task durations, and precedence relationships. Even for benchmark problems with the same number of tasks, the station configurations differ. To gain a general understanding of the problem’s behavior, the total energy consumption for each weight ratio is calculated across all benchmark problems. Table 6 provides a portion of the calculated total energy. The smallest energy value is used as a reference to determine the increase in energy consumption as the energy weight decreases during optimization. These relationships are illustrated in Fig. 3, which includes a sample of problems of varying sizes. The results indicate that energy consumption decreases significantly when it is assigned a lower weight compared to cycle time. However, the differences in total energy consumption are relatively small for weight ratios ranging from 0.2 to 1.0 but become more pronounced when optimization focuses solely on cycle time.

**Fig. 4:**
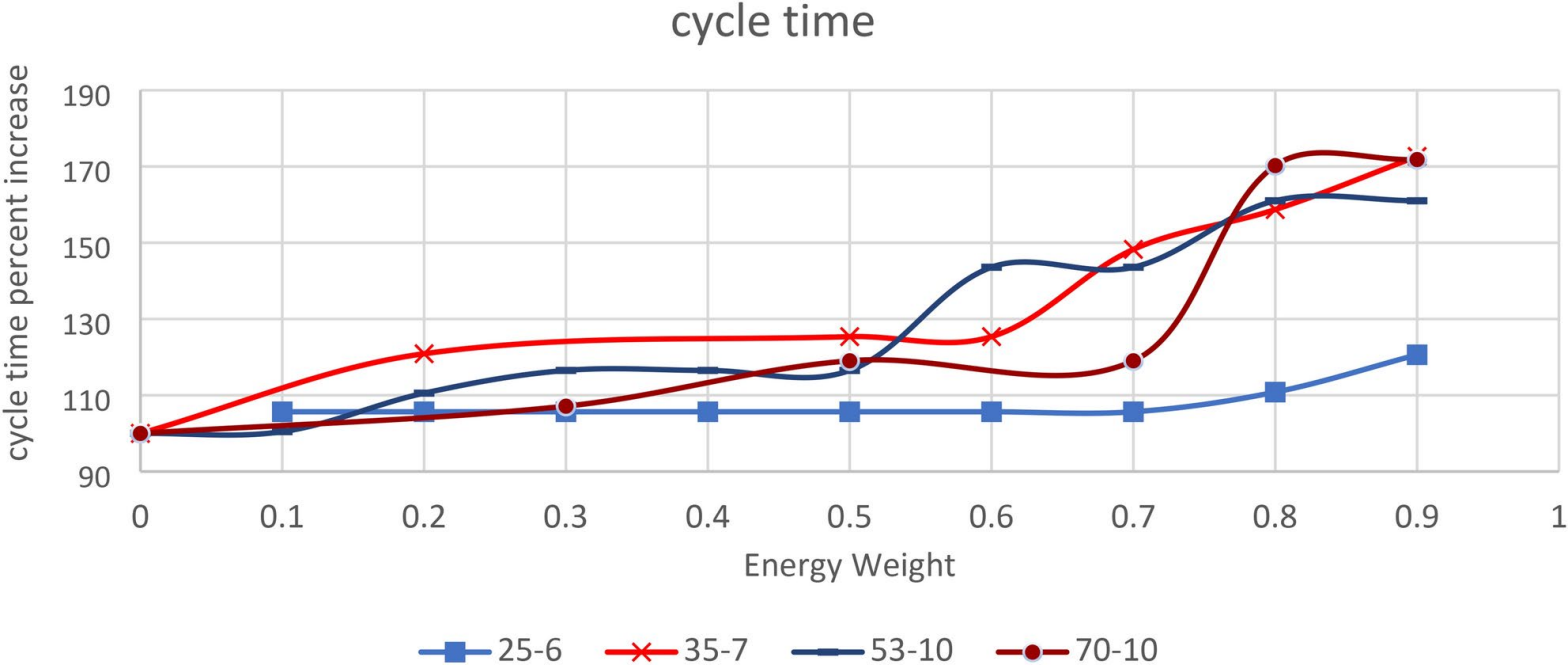
The percent increase in cycle time with energy weight increase.

Similarly, the smallest cycle time for each energy-to-cycle time weight ratio is identified, and the smallest value is used as a reference to calculate the differences in cycle time for each ratio. An example of the smallest cycle time calculation is provided in Table 7. The results, presented in Fig. 4, reveal that the differences in cycle time increase substantially as the energy weight in optimization increases. (The graphs are plotted using the percentage difference in cycle time and energy to enable comparisons across different problem sizes.)

## Conclusion

Robotic assembly lines are widely used in developed countries and are recognized for their flexibility in handling diverse product types. Given the significance of environmental concerns and the excessive costs associated with energy consumption, minimizing energy usage has become increasingly crucial in assembly line operations.

The effectiveness of the proposed method was evaluated through experiments on 17 benchmark rALB-II problems. The results revealed that standard optimization software can efficiently find optimal solutions for small-scale problems within a limited time frame. However, the proposed solution method not only achieves these optimal solutions with minimal computational effort but also proves to be efficient and effective in delivering satisfactory solutions for larger-scale problems. Furthermore, the proposed model successfully addresses multi-objective optimization, providing highly satisfactory outcomes. The results achieved using the developed model surpass those benchmark problems documented in the literature even for large size problems. The ratio of the two objectives in the objective function determines the extent of improvement in both cycle time and energy consumption. The model achieves significant energy savings even at low weights 0.2 with no significant increase in cycle time.

## Data Availability

The datasets for the benchmark problems used during current study (precedence relationships and robot task time data) was provided upon request by Zixiang Li one of the authors of paper^19^ 10.1016/j.apm.2018.08.016. While the energy data for the different types of robots are from dataset presented in paper^11^ 10.1016/j.jclepro.2014.11.041.
